# Clinical presentation of a traumatic cervical spine disc rupture in alpine sports: a case report

**DOI:** 10.1186/1757-7241-16-14

**Published:** 2008-11-12

**Authors:** Timo M Ecker, Mark Kleinschmidt, Luca Martinolli, Heinz Zimmermann, Aristomenis K Exadaktylos

**Affiliations:** 1 Department of Emergency Medicine, University of Bern, Inselspital Bern, 3010 Bern, Switzerland; 2 Department of Orthopaedic Surgery, University of Bern, Inselspital Bern, 3010 Bern, Switzerland

## Abstract

Isolated non-skeletal injuries of the cervical spine are rare and frequently missed. Different evaluation algorithms for C-spine injuries, such as the Canadian C-spine Rule have been proposed, however with strong emphasis on excluding osseous lesions. Discoligamentary injuries may be masked by unique clinical situations presenting to the emergency physician. We report on the case of a 28-year-old patient being admitted to our emergency department after a snowboarding accident, with an assumed hyperflexion injury of the cervical spine. During the initial clinical encounter the only clinical finding the patient demonstrated, was a burning sensation in the palms bilaterally. No neck pain could be elicited and the patient was not intoxicated and did not have distracting injuries. Since the patient described a fall prevention attempt with both arms, a peripheral nerve contusion was considered as a differential diagnosis. However, a high level of suspicion and the use of sophisticated imaging (MRI and CT) of the cervical spine, ultimately led to the diagnosis of a traumatic disc rupture at the C5/6 level. The patient was subsequently treated with a ventral microdiscectomy with cage interposition and ventral plate stabilization at the C5/C6 level and could be discharged home with clearly improving symptoms and without further complications.

This case underlines how clinical presentation and extent of injury can differ and it furthermore points out, that injuries contracted during alpine snow sports need to be considered high velocity injuries, thus putting the patient at risk for cervical spine trauma. In these patients, especially when presenting with an unclear neurologic pattern, the emergency doctor needs to be alert and may have to interpret rigid guidelines according to the situation. The importance of correctly using CT and MRI according to both – standardized protocols and the patient's clinical presentation – is crucial for exclusion of C-spine trauma.

## Background

Isolated non-skeletal injuries of the cervical spine are rare and among the most commonly missed injuries – with serious implications for the patient and physician[1]. In a cohort of 14,755 C-spine injuries in a level I trauma centre, Demetriades et al. showed that only 3.8% of the patients suffered from an isolated spinal chord injury without concomitant fracture or subluxation, of which only 45.5% were diagnosed as a spinal chord injury initially[1]. Specific trauma mechanisms and collateral injuries that are associated with a high incidence of skeletal C-spine injuries have been described [2]. Different algorithms for the initial assessment of these patients have been proposed, such as the NEXUS low risk criteria, or the Canadian C-spine rule[3,4]. In our institution we employ the Canadian C-spine rule as a guideline for the application of CT scans in trauma patients, since a study by Stiell et al. has proven the Canadian C-spine rule to be superior over the NEXUS criteria, especially in alert trauma patients[5]. Additional radiographic examinations, such as MRI, are important adjuncts in order to detect soft tissue injuries. However, despite rigid recommendations, emergency physicians might be challenged by situations that are rather unusual and cannot be assessed with the help of standardized scores or algorithms alone, but may require an individualized approach.

This case report shows the discrepancy between patient appearance and the extent of injury and at the same time reflects the difficulty in decision making when algorithms and guidelines are challenged by an unusual clinical presentation.

## Case report

**Figure 1:**
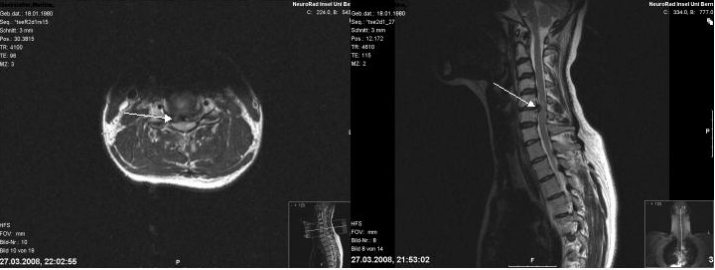
**An MRI was obtained in the emergency department for detection of disco-ligamentous injuries.** This figure shows T2 weighted transversal and sagittal MRI images. The scan revealed a traumatic extradural rupture of the intervertebral disc between C5 and C6 with ventral myelocompression but without disruption of the dorsal longitudinal ligament

**Figure 2:**
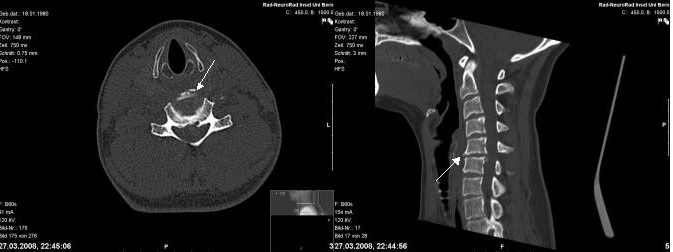
**The CT scan shows the small teardrop fracture at the ventral base plate of C5.** The ruptured disc cannot be clearly identified.

**Figure 3:**
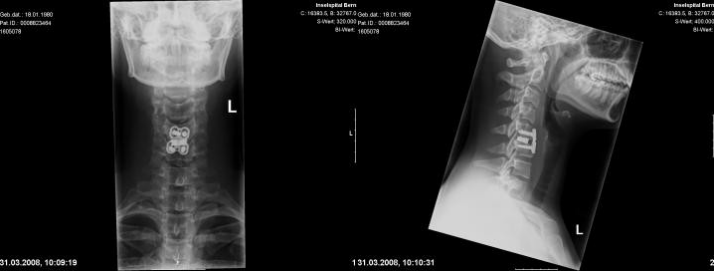
**After identification of the injury the patient was transferred to the operating room.** This figure shows the postoperative image after discectomy, cage interposition and ventral stabilization. The implants are in correct position.

We report the case of a 28-year-old female snowboarder who suffered from a fall during a descent on a maintained skiing slope. The exact mechanism of injury was not reported, but a hyperflexion injury of the C-spine was assumed. No loss of consciousness was reported. Initially the patient started to hyperventilate and was calmed by the layperson that provided the initial support. With the arrival of the emergency physician on site, the patient had a Glasgow Coma Scale (GCS) of 15 with stable hemodynamics and was subsequently transferred to our emergency department by helicopter. Upon arrival, the patient was immobilized on a vacuum mattress; the C-spine was stabilized with a Stifneck. Her GCS was 15 and primary surveys ABCDE including log roll revealed no pathologic findings. With stable vital signs, a secondary survey was performed. Since the patient was fully alert without any distracting injuries, and did not complain of any neck pain, the Stifneck was opened. Careful examination of the C-spine revealed no pain on palpation of the Proc. spinosi. She could actively turn her head to more than 45 degrees bilaterally and lift the head in a supine position without eliciting any neck pain. During the secondary survey we performed a complete neurologic exam according to the ASIA criteria. Motor function was graded according to the muscle strength scale with a score from 0 to 5 and there were no pathologic findings. The deep tendon reflexes of the upper and lower extremities bilaterally were normal. The sensory examination including light touch, vibration and pinprick, revealed a painful paraesthesia bilaterally over the palms. Applied to dermatomes the appropriate neurologic level was C6 and below. However, we did not find a complete affection of the dermatome representing the C6 level and neither of the dermatomes below this level. In the absence of cervical pain, and motor dysfunction, the underlying cause was not clear. As a differential diagnoses to C-spine trauma, tingling and paraesthesia as a consequence of the reported hyperventilation, and a peripheral nerve contusion was considered. The latter was taken into account, since she had attempted to prevent her fall with both arms extended. Subsequently, in order to safely exclude a non-skeletal injury of the spinal cord, we decided to perform an MRI. The images showed a traumatic subligamentous rupture of the intervertebral disc between C5 and C6 with ventral myelocompression (figure 1). The dorsal longitudinal ligament was intact. There was no sign of paravertebral haematoma. Consecutively, an additional CT scan was performed. The scan revealed a small teardrop fracture of the ventral base plate of C5 in the paramedian line to the left (Figure 2). The overall alignment was correct and there was no sign of myelocompression from osseous structures, nor lesions of the posterior column or the facet joints. We initiated treatment with a 30 mg/kg bolus injection of Methylprednisolone and a maintenance dose of 5,4 g/kg body weight and hour. The patient was transferred to the intermediate care unit and had surgery the next day. She underwent ventral microdiscectomy with cage interposition and ventral plate stabilization at the C5/C6 level (Figure 3). Postoperatively, the paraesthesia resolved immediately. At the time of discharge three days later, there was some residual burning and tingling, but subjective improvement of the clinical symptoms. The patient was discharged home without further complications.

## Discussion and conclusion

This case reflects several important issues. First, it confirms the findings of Franz et al.[6], who proposed that injury patterns of modern alpinists have shifted from injuries of the extremities to a higher incidence of spinal injuries. Due to the technical advances of hardware, as well as altered and more radical slope designs, snowboarding and skiing injuries have to be clearly considered high velocity accidents. Thus, as a consequence, the importance of algorithms such as the Canadian C-spine Rule has become apparent. Stiell et al.[4] have shown the associated risk between certain trauma mechanisms and the increased incidence of spinal injuries and were able to formulate important recommendations for application of CT diagnostics in such patients. We apply the Canadian C-spine Rule as a gold standard in our emergency department, since other algorithms such as the NEXUS criteria have shown to be less sensitive in the detection of injury in the alert trauma patient [5]. Especially these patients however, who are not obtunded but might have distracting injuries or might be under the influence of sedatives or pain killers, need to be evaluated according to a reliable algorithm.

Second, this case reflects the discussion in the current literature on clinical and radiographic C-spine evaluation. It seems clear that obtunded patients should be evaluated according to the C-spine protocol with an initial CT scan. Beyond this, it remains questionable which adjunct examinations should be performed. It is evident that conventional radiography is unreliable and not adequate for diagnosis of C-spine injuries, especially for evaluation of the cervico-thoracal junction [7]. Computed tomography has been shown to be the gold standard for diagnosing skeletal injury [8,9]. Stelfox et al. proved that discontinuation of C-spine immobilization after a negative CT scan is permitted and does not lead to further complications[8]. However, several authors are still discussing the importance of MRI as an adjunct. Due to its superiority in detecting disco-ligamentous injuries[3], it can be used as an adjunct examination, especially when suspecting soft tissue trauma [10-13]. The importance of MRI as an adjunct becomes apparent in our case.

In the light of different available imaging methods, this case also shows how clinical presentation and extent of injury may not be clearly associated and how deceptive the situation may appear to the emergency physician. The clinical presentation in this case was rather unusual. A patient with a cord injury typically has pain at the site of the spinal injury. This may not always be a reliable feature to exclude traumatic spinal cord injury (TSCI), since patients with TSCI often have associated brain and systemic injuries (eg, hemothorax, extremity fractures, intra-abdominal injury) that may limit the patient's ability to report localized pain. These also complicate the initial evaluation and management of patients with TSCI, and affect prognosis[14]. In this case however, we encountered a patient who was fully communicative and did not have any distracting injury. The only apparent finding was the persisting paraesthesia. The clinical presentation led us to a hesitant use of a CT scan, even though a protocol like the Canadian C-Spine rule recommends so. The indication to perform a primary MRI scan instead of a CT scan was deemed appropriate in this situation, since osseous lesions of the cervical spine were not assumed. The discrepancy between clinical presentation and MRI finding was impressive. Without the MRI and in the absence of a clinically suspicious spine, the differential diagnosis of a peripheral nerve injury might have been pursued further and the actual injury might have been missed.

Patients after high velocity accidents with suspected cervical spine injuries need to be evaluated according to strict protocols. The gold standard is the Canadian C-spine Rule. Whereas computed tomography is the gold standard for detections of skeletal injury, MRI as an adjunct is important to exclude soft tissue trauma, especially in symptomatic patients with an unsuspicious CT scan but an unclear neurologic pattern. Sometimes the clinical situation may encourage the physician to improvise and interpret guidelines to make an individual decision regarding the best imaging method to reveal the patient's pathology.

## Abbreviations

C-spine: Cervical Spine; MRI: Magnet Resonance Tomography; CT: Computed Tomography; TSCI: Traumatic Spinal Chord Injury.

## Consent

Written informed consent was obtained from the patient for publication of this case report and any accompanying images. A copy of the written consent is available for review by the Editor-in-Chief of this journal.

## Competing interests

The authors declare that they have no competing interests.

## Authors' contributions

All authors have contributed equally and sufficiently to the to conception, design and drafting and revision process of this manuscript.
